# Can an educational podcast improve the ability of parents of primary school children to assess the reliability of claims made about the benefits and harms of treatments: study protocol for a randomised controlled trial

**DOI:** 10.1186/s13063-016-1745-y

**Published:** 2017-01-21

**Authors:** Daniel Semakula, Allen Nsangi, Matt Oxman, Astrid Austvoll-Dahlgren, Sarah Rosenbaum, Margaret Kaseje, Laetitia Nyirazinyoye, Atle Fretheim, Iain Chalmers, Andrew D. Oxman, Nelson K. Sewankambo

**Affiliations:** 1 Makerere University College of Health Sciences, New Mulago Hospital Complex, Clinical Research Building, Level 4, PO Box 7072, Kampala, Uganda; 2 Norwegian Institute of Public Health, Nydalen, PO Box 4404, N-0403 Oslo, Norway; 3 University of Oslo, Blindern, Postboks 1130, 0318 Oslo, Norway; 4 Great Lakes University of Kisumu, PO Box 2224–40100, Kisumu, Kenya; 5 University of Rwanda, 101, KK 19 Av., University Avenue, PO Box: 5229, Kigali, Rwanda; 6 James Lind Initiative, Summertown Pavilion, Middle Way, Oxford, OX2 7LG UK

**Keywords:** Critical thinking, Critical appraisal, Higher order thinking, Meta-cognition, Treatment claims, Health literacy, Evidence-based health care, EBM teaching resources, Primary school curriculum, Science teaching

## Abstract

**Background:**

Claims made about the effects of treatments are very common in the media and in the population more generally. The ability of individuals to understand and assess such claims can affect their decisions and health outcomes. Many people in both low- and high-income countries have inadequate aptitude to assess information about the effects of treatments. As part of the Informed Healthcare Choices project, we have prepared a series of podcast episodes to help improve people’s ability to assess claims made about treatment effects. We will evaluate the effect of the Informed Healthcare Choices podcast on people’s ability to assess claims made about the benefits and harms of treatments. Our study population will be parents of primary school children in schools with limited educational and financial resources in Uganda.

**Methods:**

This will be a two-arm, parallel-group, individual-randomised trial. We will randomly allocate consenting participants who meet the inclusion criteria for the trial to either listen to nine episodes of the Informed Healthcare Choices podcast (intervention) or to listen to nine typical public service announcements about health issues (control). Each podcast includes a story about a treatment claim, a message about one key concept that we believe is important for people to be able to understand to assess treatment claims, an explanation of how that concept applies to the claim, and a second example illustrating the concept.

We designed the Claim Evaluation Tools to measure people’s ability to apply key concepts related to assessing claims made about the effects of treatments and making informed health care choices. The Claim Evaluation Tools that we will use include multiple-choice questions addressing each of the nine concepts covered by the podcast. Using the Claim Evaluation Tools, we will measure two primary outcomes: (1) the proportion that ‘pass’, based on an absolute standard and (2) the average score.

**Discussion:**

As far as we are aware this is the first randomised trial to assess the use of mass media to promote understanding of the key concepts needed to judge claims made about the effects of treatments.

**Trial registration:**

Pan African Clinical Trials Registry, PACTR201606001676150. Registered on 12 June 2016. http://www.pactr.org/ATMWeb/appmanager/atm/atmregistry?dar=true&tNo=PACTR201606001676150.

**Electronic supplementary material:**

The online version of this article (doi:10.1186/s13063-016-1745-y) contains supplementary material, which is available to authorized users.

## Background

The ability of individuals to obtain, process, and understand basic health information is a crucial element in making health care choices [1]. Such ability is often limited in both low- and high-income countries [2, 3]. Studies have revealed limited ability among patients, their caregivers and the public to assess the benefits and safety of treatments, and poor understanding of informed consent [4, 5], random allocation [6], risks [7, 8], and drug approval [9]. Some studies have found surprisingly low levels of knowledge of evidence-based medicine principles among health workers [10–12].

A poor understanding of health care evidence, and inadequate ability to recognise claims made about the effects of health care, can increase or diminish the willingness of individuals to seek care or participate in research. It may raise expectations (sometimes falsely), may dash hopes, or may provoke alarm (sometimes unnecessarily). It can result in low uptake of effective interventions, and inappropriate utilisation of health services [13]. It can also create communication barriers between health workers and patients [14, 15] and increase the costs of care [16, 17]. Despite these problems, there is only a handful of open-access tools to educate people to appraise information about the benefits and safety of treatments [18–22], or tools that simplify the interpretation of research information [23, 24]. To fill this gap, we have developed the Informed Healthcare Choices (IHC) resources which we are currently evaluating.

Improving access to reliable health information in the mass media – such as the Internet, radio, TV and print media – has the potential to affect individual health behaviours, health care utilisation, health care practices, and health policy [25, 26]. However, there are substantial barriers that prevent journalists from improving the scientific quality of their reports [27], and evaluations of health stories in the media have consistently found major shortcomings [28–30]. Therefore, users, listeners, viewers, and readers must be able to appraise the trustworthiness of claims in the mass media about the effects of treatments.

We have identified 32 key concepts that are relevant to evidence-informed decision-making that people need to understand and apply to assess the trustworthiness of claims made about treatment effects [31]. These concepts are grouped into six domains:

1. Recognising the need for fair comparisons of treatments
2. Judging whether a comparison of treatments is a fair comparison
3. Understanding the role of chance
4. Considering all the relevant fair comparisons
5. Understanding the results of fair comparisons of treatments
6. Judging whether fair comparisons of treatments are relevant

We have developed an educational podcast (a series of audio messages) to teach people how to apply nine of the 32 concepts in judging claims made about the effects of treatments. We settled for the nine concepts after a series of user-tests in which we identified some concepts as being more relevant than others for our target audience and easier than others for people to understand [32]. If resources allow, we plan to develop messages for other concepts, taking account of the lessons learned from this trial. The purpose of this randomised trial is, therefore, to evaluate the effects of this podcast on listeners’ abilities to assess claims made about the effectiveness and safety of treatments. We refer to this podcast as the Informed Healthcare Choices (IHC) podcast.

## Objectives

### Primary objective

To evaluate the effects of the IHC podcast on the ability of parents of primary school children to assess claims made about the effects of treatments.

### Secondary objectives

1. To evaluate the effects of the IHC podcast on listeners’ knowledge, attitudes, and intended behaviours regarding assessing claims and making decisions
2. To compare participants’ self-assessed ability to assess the reliability of claims made about treatment effects and their objective ability as measured by the Claim Evaluation Tools
3. To evaluate the effects of the IHC podcast 1-year post intervention

## Methods

### Study design

**Fig. 1:**
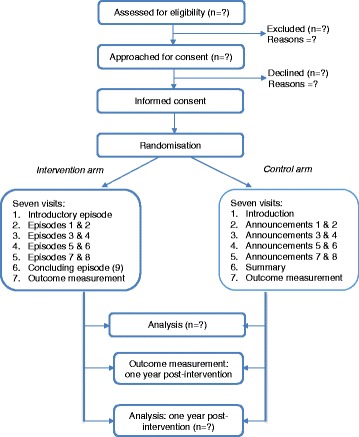
Study flow chart

This will be a two-arm, parallel-group, individual-randomised trial evaluating the impacts of a podcast series designed to teach concepts of evidence-informed decision-making to parents of primary school children in Uganda (Fig. 1).

### Setting

The study will be conducted in central Uganda within the communities in which participants live and work. Uganda is a landlocked country in East-Central Africa. It is a multiethnic country with slightly over 70% of the population aged below 25 years. The country has a life expectancy at birth of 54.9 years, total fertility rate of 5.9 children, literacy rate of 78.4%, maternal mortality ratio of 343 per 100,000 live births, and infant mortality rate of 59 per 1000 live births. It has 0.1 physicians per 1000 people [33].

#### Study population and random allocation

Our study population will be parents of primary school children in schools with limited educational and financial resources. We will draw a sample of parents of children from schools participating in a cluster-randomised trial of IHC primary school resources, designed to help primary school children understand and assess claims made about treatment effects [34].

All public and private primary schools in the central region of Uganda will be eligible to participate in the IHC primary school resources trial. The only exclusions will be international schools, special needs children’s schools for the deaf and blind, and schools or classes that participated in the user-testing and piloting of the teaching resources. We intend to include at least 120 schools in that trial.

### Inclusion and exclusion criteria

#### Inclusion

- Parents of children attending the schools participating in the trial of the IHC teaching resources
- The ability to communicate in the language of the podcast (English or Luganda)
- Consent to participate in the study

#### Exclusion

- Being unable to hear
- Not contactable by phone
- Health researchers
- Parents who have participated in the development and user-testing of the podcast
- Parents of children who participated in the development and user-testing of the children’s resources [34]

### Sampling technique

We will randomly select at least 30 schools among the 120 participating in the related trial evaluating the effectiveness of IHC materials designed for primary school children [34]. This number is informed by findings from our earlier engagements with parents, in which only about 10 to 20 parents attended meetings that are not on the usual school programme. About half of the schools selected will be from the intervention arm and the other half from the control arm of the schools participating in the trial evaluating primary school resources. In each of the selected schools we will create a list of parents of P.5 children and invite them to a meeting at the schools. At these meetings we will provide parents with information about the podcast trial and seek their consent to participate.

Because parents who attend meetings might be different than those who do not, we will also make an effort to reach parents who will not have attended the meetings through phone calls, where possible.

Parents who accept to participate will be asked to sign a Consent Form (Additional files 1 and 2) before randomisation to begin their participation in the study.

We will provide information about the trial to participants in a brochure designed as part of a package inviting parents to participate. This information will also be available on the participants’ Informed Consent Forms and will be written in the two most commonly spoken languages (English and Luganda).

### Random allocation

#### Methods for stratification and randomisation

We will stratify our sample proportionately by the highest level of education attained (primary school and below, secondary school, or tertiary education) and whether their children are in a school that is in the intervention or control arm of the IHC primary school resources trial.

We will use computer-generated blocked randomisation. A statistician or other senior researcher at the clinical trials unit at the College of Health Sciences, Makerere University who is not a member of the IHC research team will generate the allocation sequences for the two comparison groups. A web-based random number generator at www.sealedenvelope.com will be used to obtain random permuted blocks with varying block sizes of 4 and 6, and equal allocation ratios, both within blocks and within strata, as shown in Table 1 below.

**Table 1:** Excerpt from a sample randomisation list^a^

Column 1	2	3	4	5	6	7
Block identifier	Block size	Sequence within block	Allocation group	Education stratum	Number within stratum	Study code
1	4	1	Group A	≤ primary	1	YL9
1	4	2	Group A	≤ primary	2	PX5
1	4	3	Group B	≤ primary	3	JP3
1	4	4	Group B	≤ primary	4	OB2
2	4	1	Group A	≤ primary	5	XD6
2	4	2	Group A	≤ primary	6	JD0
2	4	3	Group B	≤ primary	7	NU5
2	4	4	Group B	≤ primary	8	SS0
3	4	1	Group B	≤ primary	9	SK0
3	4	2	Group A	≤ primary	10	QN4

^a^List generated from www.sealedenvelope.com

As shown in Table 1 above, the list will have study codes that are unique for each participant (column 7), against which we will attach an ordered array of numbers (column 6) spanning the full breadth of our sample size within each stratum. Each study code will be printed on a separate opaque envelope.

#### Methods for allocation concealment

The allocation group corresponding to each study code will be printed on a separate slip, inserted and sealed in the corresponding opaque envelope. As shown in the list above, the first participant to be recruited among those with primary or no education will have the study code YL9 on their envelope, inside of which will be a small note with their allocation group (group A).

To reduce the risk of bias during allocation a separate list containing the study participant number, and corresponding study code and number within each stratum, will be generated for each of the three education-level strata (up to and including primary, at least secondary and tertiary), as shown in Table 2 below. This list – which will not identify the study arm – will be given by the statistician to a research assistant who will be in charge of group allocation.

**Table 2:** Sample participant recruitment lists for each stratum

Stratum 1: Primary school and below		Stratum 2: At least secondary school		Stratum 3: At least tertiary education	
Number within stratum	Study code	Number within stratum	Study code	Number within stratum	Study code
1	YL9	1	FO4	1	TH4
2	PX5	2	HG6	2	UW0
3	JP3	3	GS3	3	AT5
4	OB2	4	PS9	4	SX4
5	XD6	5	YE9	5	UJ7
6	JD0	6	BH7	6	XD9
7	NU5	7	HN6	7	RJ7
8	SS0	8	TY7	8	CS1
9	SK0	9	DT3	9	BC4
10	QN4	10	FZ1	10	ED1

Upon completion of procedures for confirming consent of a willing participant the research assistant responsible for recruiting the participant will call the research assistant in charge of group allocation. The research assistant responsible for allocation will then open the next available envelope in the stratum, corresponding to the participant’s education level and study arm of the child’s school, to determine the study group to which the parent will be allocated.

### The interventions

Participants in the intervention group will listen to a series of podcast episodes designed to teach people to assess claims made about treatment effects (the IHC podcast). Each episode includes a short story with an example of a treatment claim, a simple explanation of a concept used to assess that claim, another example of a claim illustrating the same concept, and its corresponding explanation. In each story there is a question about the trustworthiness of a claim, which is resolved by applying the relevant key concept. All episodes have a conclusion with a take-home message emphasising the concept of evidence-informed, health decision-making. The examples used in the podcast are for claims made about treatments for health conditions, which are of interest to the target audience, such as malaria, diarrhoea, and HIV/AIDS. We have also included claims made about some common practices, such as contraception, which are of interest to our audience.

The topics and claims were identified from scanning recent mass media reports and interviewing parents [32]. There are eight main episodes in the series covering the nine core concepts (Table 3). Each episode lasts about 5 min. One of the episodes (episode 1) covers two closely related key concepts (1.1 and 5.1 in Table 3). Two additional episodes introduce the podcast and summarise the key messages from the first eight episodes, respectively. The podcast also has a song (the IHC theme song), sections of which play at the beginning, in the background and at the end of the episodes. The lyrics of the song were designed to reinforce the messages of the podcast and focus on being careful about claims, and the important questions to ask when we hear claims made about treatment effects. The final structure, content, presentation of the content in each episode, and the series as a whole was informed by an iterative user-centred process of development and user-testing described in another paper [32]. This process involved consultation with various stakeholders, including parents in our target audience, on the appropriate content to be included, and the presentation of this content in each episode, and in the podcast as a whole [32]. The numbering in the first column in the table below relates to numbering of the key concepts in their respective domains, as presented by Austvoll-Dahlgren et al. [31].

**Table 3:** The nine concepts^a^ included in the IHC podcast

Concepts	Explanations	Implications
1.1 Treatments may be harmful	People often exaggerate the benefits of treatments and ignore or downplay potential harms. However, few effective treatments are 100% safe	Always consider the possibility that a treatment may have harmful effects
1.2 Personal experiences or anecdotes (stories) are an unreliable basis for assessing the effects of most treatments	People often believe that improvements in a health problem (e.g. recovery from a disease) were due to having received a treatment. Similarly, they might believe that an undesirable health outcome was due to having received a treatment. However, the fact that an individual got better after receiving a treatment does not mean that the treatment caused the improvement, or that others receiving the same treatment will also improve. The improvement (or undesirable health outcome) might have occurred even without treatment	Claims made about the effects of a treatment may be misleading if they are based on stories about how a treatment helped individual people, or if those stories attribute improvements to treatments that have not been assessed in systematic reviews of fair comparisons
1.3 A treatment outcome may be associated with a treatment, but not caused by the treatment	The fact that a treatment outcome (i.e. a potential benefit or harm) is associated with a treatment does not mean that the treatment caused the outcome. For example, people who seek and receive a treatment may be healthier and have better living conditions than those who do not seek and receive the treatment. Therefore, people receiving the treatment might appear to benefit from the treatment, but the difference in outcomes could be because of their being healthier and having better living conditions, rather than because of the treatment	Unless other reasons for an association between an outcome and a treatment have been ruled out by a fair comparison, do not assume that the outcome was caused by the treatment
1.4 Widely used treatments or treatments that have been used for a long time are not necessarily beneficial or safe	Treatments that have not been properly evaluated but are widely used or have been used for a long time are often assumed to work. Sometimes, however, they may be unsafe or of doubtful benefit	Do not assume that treatments are beneficial or safe simply because they are widely used or have been used for a long time, unless this has been shown in systematic reviews of fair comparisons of treatments
1.6 Opinions of experts or authorities do not alone provide a reliable basis for deciding on the benefits and harms of treatments	Physicians, researchers, patient organisations, and other authorities often disagree about the effects of treatments. This may be because their opinions are not always based on systematic reviews of fair comparisons of treatments	Do not rely on the opinions of experts or other authorities about the effects of treatments, unless they clearly base their opinions on the findings of systematic reviews of fair comparisons of treatments
2.1 Evaluating the effects of treatments requires appropriate comparisons	If a treatment is not compared to something else, it is not possible to know what would happen without the treatment, so it is difficult to attribute outcomes to the treatment	Always ask what the comparisons are when considering claims made about the effects of treatments. Claims that are not based on appropriate comparisons are not reliable
2.2 Apart from the treatments being compared, the comparison groups need to be similar (i.e. ‘like needs to be compared with like’)	If people in the treatment comparison groups differ in ways other than the treatments being compared, the apparent effects of the treatments might reflect those differences rather than actual treatment effects. Differences in the characteristics of the people in the comparison groups might result in estimates of treatment effects that appear either larger or smaller than they actually are. A method, such as allocating people to different treatments by assigning them random numbers (the equivalent of flipping a coin), is the best way to ensure that the groups being compared are similar in terms of both measured and unmeasured characteristics	Be cautious about relying on the results of nonrandomised treatment comparisons (for example, if the people being compared chose which treatment they received). Be particularly cautious when you cannot be confident that the characteristics of the comparison groups were similar. If people were not randomly allocated to treatment comparison groups, ask if there were important differences between the groups that might have resulted in the estimates of treatment effects appearing either larger or smaller than they actually are
4.1 The results of single comparisons of treatments can be misleading	A single comparison of treatments rarely provides conclusive evidence and results are often available from other comparisons of the same treatments. These other comparisons may have different results or may help to provide more reliable and precise estimates of the effects of treatments	The results of single comparisons of treatments can be misleading
5.1 Treatments usually have beneficial and harmful effects	Because treatments can have harmful effects as well as beneficial effects, decisions should be informed by the balance between the benefits and harms of treatments. Costs also need to be considered	Always consider the trade-offs between the potential benefits of treatments and the potential harms and costs of treatments

^a^From Austvoll-Dahlgren et al. [31]

A summary of the concepts contained in each episode of the podcast is shown in Table 4 below.

**Table 4:** The concepts^a^ contained in each episode of the podcast

Episode	1	2	3	4	5	6	7	8
Concept^a^	1.1 + 5.1	2.1	1.2	1.3	1.4	1.6	2.2	4.1

^a^See Table 3 for the full description of the concepts and their implications

Participants in the control group will receive typical public service announcements about conditions of interest to our target audience, including malaria, HIV/AIDS, and diarrhoea, that have been included in the podcast. These audio messages are already being aired or have in the recent past been aired on radio. They are freely available and will be sourced from radio stations and media houses involved in disseminating information from the Ministry of Health.

### Delivery of the interventions

The interventions will be delivered over a period of 7 to 10 weeks to participants in the communities where they live or work. At least 20 research assistants will help in the field recruitment, administration of the intervention, follow-up of participants in the community, and administration of the outcome assessment tool. Each research assistant will be allocated about 25 participants to follow up through the duration of the study. The research assistants will visit all their allocated participants every week, delivering the interventions on portable media players to an average of four participants per day. In this study setting this approach is currently the most practical and most reliable way of delivering the interventions for this trial.

**Fig. 2:**
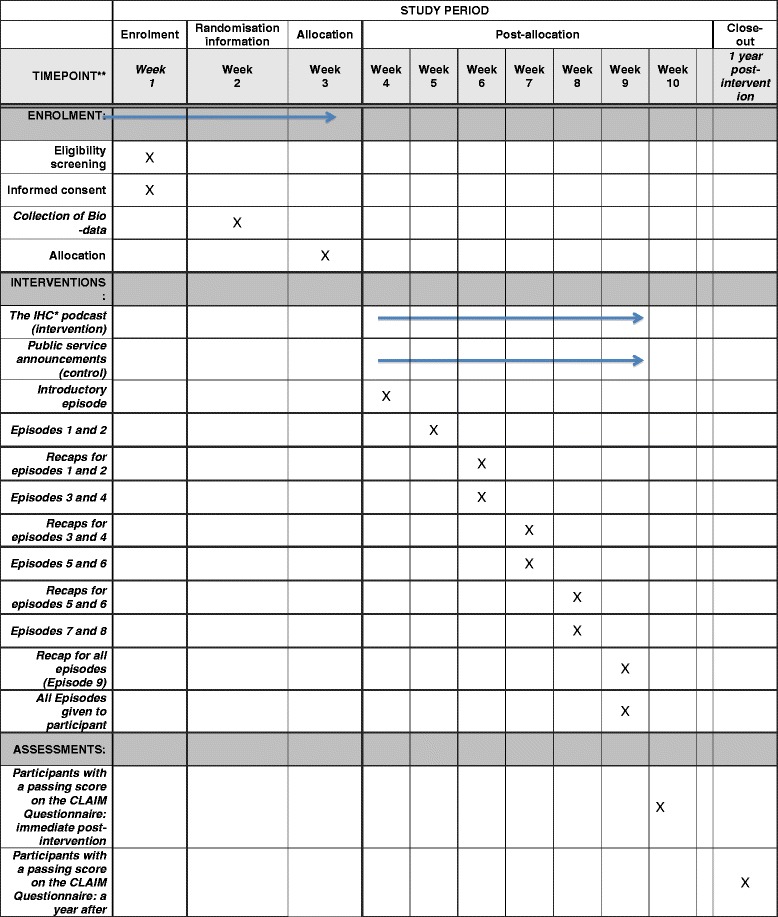
Schedule of enrolment, interventions, and assessments

One-time brief interventions have not been shown to improve knowledge of evidence-based medicine principles [35, 36] among health workers. So, as part of our intervention, at every visit we will play a recap of the previous episodes before playing the new episodes. Each participant will receive at least one supervised listening session of every episode, delivered by the research assistants over 7 to 10 weeks. In addition to the messages delivered by the research assistants, we will provide participants with the complete podcast on MP3 players, so that they can replay the episodes at their convenience. For the intervention group we will start with simpler concepts and gradually introduce more difficult concepts as the podcast series progresses, as summarised in Table 5 below. For the control group we will introduce episodes with health conditions that are similar to those being delivered in the intervention group at a defined period of time. The timing of study procedures is summarised in Fig. 2 below.

**Table 5:** Sequence of delivery of the episodes of the podcast

Week/Visit	1	2	3	4	5	6	7
Episode	Intro	1 + 2	3 + 4	5 + 6	7 + 8	9	Evaluation^a^

^a^Study evaluation tool (Additional file 3).

### Outcome assessment

We will use a modified version of the Claim Evaluation Tools as the outcome measure [37]. The Claim Evaluation Tools consist of multiple-choice questions that assess an individual’s ability to apply 32 concepts that people need to be able to understand and apply to assess the validity of treatment claims and to make informed health care choices. In addition, it includes questions that assess intended behaviours, self-efficacy, attitudes, and satisfaction. Development of the Claim Evaluation Tools has been based on extensive qualitative and quantitative feedback from methodological experts, health professionals, teachers, and members of the public. We have conducted psychometric testing and Rasch analysis with a diverse sample of 1000 people, including P.5 children exposed to pilot versions of the IHC primary school resources, children who were not exposed, adults with little or no exposure to the concepts, and adults who are familiar with the concepts. The evaluation tool was found to have high reliability (Cronbach’s alpha 0.81), and to be unidimensional (there was no evidence of subdimensions measuring different traits). Furthermore, there was weak or no dependence between items (no items were redundant). Results from the pilot revealed that parents in the intervention group performed slightly better compared with those in the control group. Many of the participants in the pilot preferred Luganda to English and had difficulty reading the questions in English.

After removing some questions, we modified the remaining questions that did not perform well according to the Rasch analysis, and revised and simplified the text where needed. The items were also translated into Luganda and adapted for audio administration. In the second psychometric testing, the Claim Evaluation Tools were administrated to a similar sample as described in the first psychometric test, but approximately half of the sample received the items as a written questionnaire in English and the other half received the Luganda audio versions. The results of this test suggested that the items administered in English performed very well according to the Rasch model, and with high reliability. Furthermore, the items were also less difficult than what was found in the first psychometric testing before the revisions. The results also suggested that the Luganda versions of items had evidence of under-discrimination and differential item functioning in seven out of 29 items. These items were revised to improve fit to the Rasch model.

Based on these two psychometric tests, a selection of 18 multiple-choice items addressing the nine concepts that the IHC podcast covers will be used (Additional file 3). Each key concept is evaluated by two items. We chose items with high reliability (fit to the Rasch model) and those with an appropriate difficulty level.

Parents in both arms of the trial will complete the questionnaires after they have listened to the podcast or public service announcements. Research assistants will deliver the questionnaires, ensuring that the parents have adequate time to answer the questions and ensure that the questionnaires have been completed fully.

### Absolute standard (for passing scores)

We will use an absolute (criterion referenced) standard to set a passing score for the version of the Claim Evaluation Tools that we will use, i.e. based on how much the parents know and are able to apply. Parents will be counted as ‘passing’ or ‘failing’ depending on whether they meet a specified criterion. We used a combination of Nedelsky’s [38] and Angoff’s [39] methods to determine the criterion, which is a cut-off for a passing score, as described in Additional file 4. In addition, we determined a second cut-off for a score that indicates mastery of the nine concepts, using the same methods, as described in Additional file 4. The criterion for passing is a minimum of 11 out of 18 questions answered correctly. The criterion for mastery is a minimum of 15 out of 18 questions answered correctly.

### Primary outcomes

1. The difference between the intervention and control groups in the proportion of participants with a passing score
2. The mean difference in the score (number of correct answers) for all of the questions that assess their ability to apply the nine concepts that are included in the IHC podcast

### Secondary outcomes

1. The difference between the intervention and control groups in the proportion of parents with a score indicating mastery of the concepts (see above)
2. The difference between the intervention and control groups for each concept
3. Differences in attitudes and intended behaviours between the two groups

The intervention and control groups will complete the Claim Evaluation Tools after listening to all of the IHC episodes or public service announcements, respectively. We will evaluate the effects of the IHC podcast series again after 1 year, using the same outcome measures. We will try to locate participants who will have moved from their original residence or work place and document those who are lost to follow-up, as well as the reasons, where possible.

### Blinding

Because of the nature of the intervention, the research assistants who will deliver the intervention, the principle investigator supervising them, and the participants in the study will not be blinded. The statistician who will analyse the study results will not be told which group is the intervention group until after the analyses are completed.

To ensure uniform performance in delivery of the intervention and assessment of outcomes, all study staff will receive joint training before the start of the trial and will receive refresher training in the course of the trial. We will have standard operating procedures to guide interactions with participants.

### Data collection and management

#### Postrecruitment retention strategies

We will maintain a database of participants’ contact details and maps to their homes to enable follow-up. The research assistants will keep a study logbook and use a progress tracking checklist (Tables 6 and 7) to keep track of their progress and note any problems with follow-up of participants. We will discuss these problems and ways of addressing them and preventing loss to follow-up at weekly meetings.

**Table 6:** Study log for monitoring progress of the intervention for each study participant

Study number _____________			Date enrolled ____________	Study arm _____
Visit number	Date contacted by phone	Date seen	Date intervention was administered	Observations and comments for study visit
ᅟ	ᅟ	ᅟ	ᅟ	ᅟ
ᅟ	ᅟ	ᅟ	ᅟ	ᅟ
ᅟ	ᅟ	ᅟ	ᅟ	ᅟ

**Table 7:** Sample study procedures checklist for all participants

Research assistant: ________________ Contacts________ Date started_________											
	Participant particulars			Dates for study procedures and visits							
ID	Name	Group	Contacts	Consent	Intro	1–2	3–4	5–6	7–8	9	Claim Evaluation Tool
ᅟ	ᅟ	ᅟ	ᅟ	ᅟ	ᅟ	ᅟ	ᅟ	ᅟ	ᅟ	ᅟ	ᅟ
ᅟ	ᅟ	ᅟ	ᅟ	ᅟ	ᅟ	ᅟ	ᅟ	ᅟ	ᅟ	ᅟ	ᅟ
ᅟ	ᅟ	ᅟ	ᅟ	ᅟ	ᅟ	ᅟ	ᅟ	ᅟ	ᅟ	ᅟ	ᅟ

### Data collection and management

We will use paper questionnaires for the outcome assessment and we will collect baseline sociodemographic characteristics using a paper form. For participants who prefer listening to the Luganda version of the podcast but are unable to read Luganda we will use an interviewer-administered, standardised Luganda audio version of the evaluation tool. A research assistant will enter their responses to the audio evaluation tool on a paper questionnaire. To reduce any wrong entries and missing values the research assistants will check the questionnaires for completeness and for unclear responses immediately after completion. We will use Epidata version 3 software [40] to enter all data from the paper questionnaires and convert them into an electronic dataset. To reduce errors during data entry we will double-enter all the data. All data entrants will be trained on the meaning of variables and how they should be entered into the database. Questions that remain unanswered will be scored as ‘wrong’. The lead investigator will double-check the entries.

All copies of Consent Forms will be kept separate from the completed questionnaires and baseline characteristics of the participants. Both signed Informed Consent Forms and completed questionnaires will be kept under lock and key. All electronic data will be password-protected and kept on a separate hard disc, which will be backed up every week to prevent data loss. The hard discs will also be kept under lock and key and access will be restricted to study investigators.

Trial progress reports will be compiled by the study team and submitted to the regulatory bodies (Uganda National Council for Science and Technology and School of Medicine Research and Ethics Committee (SOMREC), Makerere University College of Health Sciences), in accordance with their respective guidelines. Final project reports (including trial reports) will be compiled by the study staff and submitted to the relevant bodies (Research Ethics Committees (RECs) and funders), as per their requirements.

### Analysis

We will present participants’ age, sex, and highest level of education attained as frequencies and proportions.

For the primary and secondary outcomes, we will use fixed-effect models with the stratification variable (education) modelled as a fixed effect, using logistic regression for dichotomous outcomes, linear regression for continuous outcomes and Poisson regression for count outcomes. For the questions that assess applied knowledge or understanding, missing values will be counted as ‘wrong’ answers.

We will report the proportion (for dichotomous outcomes), mean and standard deviation (for continuous outcomes), or median and interquartile range (for count outcomes) in each group, the estimated difference, the estimated confidence interval for the difference, and the *P* value for the difference between groups from the statistical model for each of the primary and secondary outcomes. For attitudes and intended behaviours (Additional file 3), we will dichotomise each outcome by combining, for example, ‘very unlikely’ with ‘unlikely’ and as ‘likely’ with ‘very likely’; and we will report the proportion in each of the four categories.

#### Subgroup and exploratory analyses

Education might affect people’s ability to understand and apply the concepts and to answer questions in the Claim Evaluation Tools. We will explore whether there are differences in the effects of the intervention for participants with primary school education versus secondary school education versus tertiary education. We will conduct tests for interaction for the primary outcomes, and we will use published guidelines to interpret the results of these subgroup analyses [41], using the models described above.

The children of all participants will be participating in a parallel trial evaluating the effects of IHC teaching resources, designed to teach P.5 school children concepts that they need to understand and apply to assess treatment claims [34], but only half of them will be receiving the IHC teaching resources. By testing for interaction between IHC teaching resources and the podcast in the statistical models described above we will evaluate whether the combination of the podcast and the IHC teaching resources improves outcomes among parents, compared to the podcast alone.

### Sample size

We used the method described by Donner [42] to calculate the sample size, based on calculation of odds ratios. The smallest difference between the intervention and control groups that we want to be able to detect in the proportion of respondents with a passing score is 10 percentage points. Assuming that 10% of the control group will achieve a passing score (a conservative estimate, based on data from the pilot [32], power of 0.90, a two-sided *P* value of 0.05, we estimate that 397 participants will be needed to be able to detect an improvement of 10% in the intervention group. Studies of the effects of drug fact boxes and a primer to help people understand risk suggest that this is likely to be an adequate sample size [25, 26]. Allowing for a 20% loss to follow-up gives us a sample size of 497 participants. More specific information about the trial and this protocol is summarised in the SPIRIT checklist (Additional file 5).

### Safety monitoring and adverse events

The National Council of Science and Technology in Uganda has given this study a very low rating for risk to participants. Nonetheless, we will monitor unexpected adverse events and problems that might arise.

### Stakeholder involvement

We have engaged health researchers, journalists, other media and communications practitioners, teachers, and the general public in developing, revising, piloting, and user-testing the podcast [32].

### Reporting, dissemination and notification of results

Authorship of publications arising from this study will be according to contribution. Publications and the resources will be open access, allowing free noncommercial use, distribution, reproduction and further development of the work provided the source is properly cited. We will acknowledge all staff participating in the trial.

The results of this trial will be publicised through a variety of channels. All of the resources will be made available on the IHC project and Testing Treatments interactive websites, and through the Critical Appraisal Resource Library (CARL). They will be disseminated internationally by the IHC project team and through members of our international advisory group, the Cochrane Collaboration (www.cochrane.org), the Evidence-informed Policy Network (www.evipnet.org), the WHO, the Campbell Collaboration (www.campbellcollaboration.org), and other relevant networks and organisations.

## Discussion

To the best of our knowledge this is the first randomised trial to assess the use of mass media to promote understanding of the key concepts that are essential to improving people’s ability to critically assess claims made about the effects of treatments. A basic understanding of these concepts is envisaged to improve critical assessment of things that people say about what effects treatments might have if, or when, we use them.

In this context the factors, which may facilitate or hinder people’s understanding of information on assessing claims made about treatment effects, are largely unknown. Therefore, we plan to conduct a process evaluation to explore these and any other issues that might explain why the intervention worked or did not work as expected. We shall use this information to improve the design and administration of future interventions of this nature.

It is expected that recruitment of parents will be slow and follow-up may be difficult. From earlier pilots done in this context, very few parents attended parents’ meetings at schools and those who did arrived very late. The majority of our study participants are very likely to be women since mothers tend to be the ones who commonly attend parents’ meetings. It is not known how representative these will be of our intended study population. In our process evaluation we shall explore reasons why, if this turns out to be the case, and reasons for choosing not to participate among those who decline to consent.

### Trial status

This study is currently recruiting participants.

## Additional files

Additional file 1: Consent form Podcast trial English version. (DOCX 27 kb)
Additional file 2: Consent form Podcast trial Luganda translated version. (DOCX 29 kb)
Additional file 3: The CLAIM Evaluation Tools. (ZIP 1007 kb)
Additional file 4: Setting a standard for the “Claim 12” and “Claim 9”. (DOCX 28 kb)
Additional file 5: SPIRIT 2013 Checklist: Recommended items to address in a clinical trial protocol and related documents*. (DOC 122 kb)

## Abbreviations

AIDS: Acquired immunodeficiency syndrome
CARL: Critical Appraisal Resource Library
HIV: Human immunodeficiency virus
IHC: Informed Healthcare Choices
P. 5: Primary five
REC: Research and Ethics Committee
SOMREC: School of Medicine Research and Ethics Committee
UNCST: Uganda National Council for Science and Technology
WHO: World Health Organisation
