# Supplementary material for: Can an educational podcast improve the ability of parents of primary school children to assess the reliability of claims made about the benefits and harms of treatments: study protocol for a randomised controlled trial
Source: Trials. 2017 Jan 21;18:31. doi: 10.1186/s13063-016-1745-y (PMC5251251; doi:10.1186/s13063-016-1745-y)
Supplement: Additional file 1: — Consent form Podcast trial English version. (DOCX 27 kb) [file 13063_2016_1745_MOESM1_ESM.docx]

**Additional file 1. RESEARCH PARTICIPANT INFORMED CONSENT FORM-ENGLISH**

**INTRODUCTION**

The Informed Healthcare Choices Project is a collaborative research project that aims to improve the ability of people to assess health information and make better health choices. This research study is being conducted by researchers from Makerere University College of Health Sciences and researchers from other countries.

As part of this research study we have worked with health researchers, journalists, teachers, children and other members of the public to develop a series of audio messages that we think could teach people how to assess health information and make informed healthcare choices. We would like to find out if these audio messages actually improve people’s understanding and ability to assess information about treatments.

The purpose of this form is to request your participation in this research study. Below is some information about this study that you might need before you can make this decision. It is important for you to go through it and understand it before you agree to participate.

1. **WHY IS THIS research BEING CONDUCTED?**

To find out if the audio messages prepared by the Informed Healthcare Choices project team improve the way people understand and assess information about treatments.

1. **HOW will ThIS study be conducted?**

We shall approach parents like you, of primary school children in central Uganda and ask them to participate. Those who accept to participate will be randomly divided into two groups: one group to receive messages that are prepared by the Informed Healthcare Choices project and another group to receive messages that are prepared by the Ministry of Health to teach people about diseases and their treatments. There are 9 messages in total and it takes about 5 minutes to listen to each message.

If you agree to participate, a member of our research team will visit you about seven times. That person will play two messages for you during each of the first six visits, and will play a recap of the messages you listened to the previous week, before playing the new messages if you so wish. You will also be given a portable audio player to enable you to listen to the messages again at your convenience.

At the last visit, after you have listened to all the messages, we will ask you to complete a short questionnaire. The answers you provide will enable us understand if the messages you listened to helped you understand how to assess health information. After the last visit you may be contacted again for feedback and your experiences having participated in the trial. This information will help us understand why the podcast worked or did not work (the process evaluation of the trial). One year after completing this questionnaire we will visit you again and ask you to complete the same questionnaire again to see if you still remember what you learned from the messages. Overall our interactions with you will be friendly and each visit will last about 30 minutes.

1. **Are there any risks to you?**

Your participation in the study presents no known risks to you. However, it will require some of your time.

1. **WHAT WILL YOU benefit FROM PARTICIPATING IN THE STUDY?**

You might gain some new knowledge about treatments and how to make informed healthcare choices. There will be no other direct benefit to you from participating in this study.

1. **Will you incur any costs?**

You will not incur any costs as a result of taking part in the study, other than the time taken to participate.

1. **Will you be rewarded for participating in the study?**

You will not gain any form of reward, monetary or otherwise from participating in this study.

1. **will we share the information you give us with other people?**

The information you provide to us will be kept confidential in accordance with the ethical standards agreed upon by the local and international organizations governing the conduct of research involving human participants. Information from this study will not reveal your identity.

1. **YOUR Rights to Refuse/Withdraw From the study**

Your participation in this research is purely voluntary and you are free to decline to take part or withdraw at any time without any repercussions.

1. **Questions ABOUT THE RESEARCH**

In case of any further questions, please contact Professor Nelson K. Sewankambo, the Principal Investigator at Makerere University College of Health Sciences, P.O. Box 7072, Kampala Uganda: Tel: 0414 530021 or Dr. Daniel Semakula and Ms. Allen Nsangi, co-investigators on 0773333629 or 0776543000. If you have questions regarding the ethics of this research, you may contact Prof. James Tumwine, Chairperson, MakCHS School of Medicine Research and Ethics Committee. Tel: 0414 531875.

1. **participant’s DECLARATION OF Consent**

I have understood the above information about this research study and all my questions have been answered. I feel that I have been given enough information and time to consider my decision to join the study. I fully understand that by signing this form, I do not waive any of my legal rights, nor does it relieve the study investigators of their liability. By signing this form, I confirm that I have been informed about the research study in which I am voluntarily agreeing to take part. A copy of this form has been given to me.

Having understood all the information pertaining to this study I therefore agree to my participation in this study by appending my signature and name below.

| **Research Participant**  Name:_____________________________ | Signature: _________________________ |
| --- | --- |
| Date:______________________________ | Tel number:________________________ |

| **Witness**  Name:____________________________ | Signature: ________________________ |
| --- | --- |
| Date: ____________________________ | Tel number: _______________________ |
