# Supplementary material for: Can an educational podcast improve the ability of parents of primary school children to assess the reliability of claims made about the benefits and harms of treatments: study protocol for a randomised controlled trial
Source: Trials. 2017 Jan 21;18:31. doi: 10.1186/s13063-016-1745-y (PMC5251251; doi:10.1186/s13063-016-1745-y)
Supplement: Additional file 2: — Consent form Podcast trial Luganda translated version. (DOCX 29 kb) [file 13063_2016_1745_MOESM2_ESM.docx]

**Additional file 2. EKIWANDIIKO EKIKUSABA OLUKUSA OKWETABA MUKUNOONYEREZA**

**(RESEARCH PARTICIPANT INFORMED CONSENT FORM-LUGANDA)**

**ENYANJULA.**

Pulojekiti ya “Supporting Informed Healthcare Choices in Low-Income Countries (SIHCLIC)” yatandikibwawo okunoonyereza ku ngeri gyetuyinza okuyambamu abantu baffe okutegeera, okwekebejja n’okusunsula obubaka obukwata ku by’obulamu, olwo basobole okukola okusalawo okulungi ku nsoga z’obulamu bwabwe. Okunoonyereza kuno kukolebwa abakugu okuva mu tendekero ly’abasawo abakugu mu “Makerere University” (Makerere University College of Health Sciences) awamu n’abakugu abalala aokuva munsi endala.

Mukunoonyeresza kuno tukolaganye ne banamawulire, abasawo abakugu mukunyereza, abaana abato, abasomesa n’abantu abalala abaabulijjo, mukukola obubaka obwenjawulo obukwata ku by’obujanjabi. Tusuubira nti obubaka buno busobola okuyigiriza abantu okukola okusalawo okulungi kunsonga z’ebyobujjanjabi. Twagala okumanya oba nga ddala obubaka buno buyigiriza abantu ku by’okusalawo ku by’obujjanjabi.

Ebbaluwa eno ekusaba weetabe mu kunoonyereza kuno. Wammanga tukwanjulidde ebintu by’olina okutegeera nga tonasalawo kwetaba mukunonyereza kuno era tukusaba n’obuwombeefu obyekkaanye nga tonnassa mukono kukiwandiiko kino.

1. **oKUNOONYEREZA KUNO lWAKI KUKOLEBWA?**

Okunoonyereza kuno tukukoze kutuyambe okulaba oba nga ddala obubaka bwetuteeseteese buyigiriza abantu okukola okusalawo okulungi kuby’obujjanjabi oba nedda.

1. **oKUNOONYEREZA KUNO KUNAAKOLEBWA KUTYA?**

Okunoonyereza kuno kujja kwetabwamu abantu abenjawulo. Abazadde abalina abaana mu masomero ga “primary.” Tujja kutuukirira abazadde nga gwe abalina abaana mumasomero ga “primary”, tubasabe okwetaba mukunoonyereza kuno. Abo abanakkiriza baggya kugabanyizibwamu ebiwayi bibiri. Ekiwayi ekimu kijja kufuna era kiwulirize obubaka ku by’obulamu obukoleddwa project eno ate ekiwayi ekirala kyo kijja kufuna obubaka ku by’obulamu obwabulijjo obukolebwa Minisitule Y’ebyobulamu bwetuwulira ku maradio bulijjo. Obubaka buno buli mubitundutundu mwenda (9), era nga buli kitundu kya dakiika nga taano (5).

Singa obeera okkirizza okwetaba mu kunoonyereza kuno, tujja kukuba akalulu okosobola okulaba kiwayi ki ky’onoogwamu. Oluvannyuma ojja kuweebwa obumu kububaka bwetukutegeezezza waggulu awo okusinziira ku bkiwayi ky’onoogwamu. Omu kubantu baffe ajja kukukyalira emirundi musanvu (gwe mulundi gumu mu wiiki okumala wiiki nga musanvu). Buli mulundi gw’onookyalirwa ojja kuweebwa obubaka kuby’obulamu obuwulirize. Omulundi ogunaasembayo ojja kuweebwayo ebibuuzo. Ebibuuzo bino bijja kutuyamba okumanya oba nga ebintu by’osomeseddwa mububaka pbwenjawulo bisobola okusomesa omuntu okukola okusalawo okulungi ku by’obujanjabi. Obubaka bwonna bwonawuliriza tujja kubukulekera ku “karadio” akanaakusobozesa okubuwuliriza buli lwoba oyagadde. Era oluvannyuma ttujja kukubuuzaayo ebibuuzo ebirala ebinaatutegeeza lwaki obubaka buno buayambye oba tebuyambye.

Nga wayiseewo ebbanga lya mwaka gumu tujja kukomawo tukubuuze ebibuuzo byebimu tulabe oba nga obubaka obwo onooba okyabujjukira nga wayiseewo ebbanga lya mwaka.

1. **WALIWO OBULABE BWONNA OBUYINZA OKUKUTUUKAKO?**

Nedda, tewali bulabe bwonna bujja kukutuukako olw’okwetaba mukunoonyereza kuno. Wabula kisoboka nti obudde bwotuwa bwandikukosaamu katono.

1. **KIKI KY’ONOOFUNAMU MUKWETABA MUKUNOONYEREZA KUNO?**

Tusuubira nti bw’oneetaba mukunoonyereza kuno osobola okuyiga ebintu ebyenjawulo ku by’obulamu.

1. **Weetaagisibwa okusasula OKUBEERA MUKUNOONYEREZA KUNO?**

Nedda! Teweetaaga kusasula nusu yonna okusobola okwetaba mukoneereza kuno.

1. **Onosasulwa olw’okwetaba mukunoonyereza kuno?**

Tojja kusasulwa mungeri yonna olw’okwetaba mukunoonyereza kuno.

1. **EBINTU BY’OTUBUULIRA TUNAABIBUULIRA ABANTU ABALALA?**

Nedda! Ebintu byotubuulira byonna tujja kubikuuma nga bya kyama, nga amateeka g’ensi yonna agafuga ebyokunoonyereza bwegatugamba.

1. **Eddembe lyo okugaana n’okuva mukunoonyereza kuno.**

Okwetabaakwo mukunoonyereza kuno kwa kyeyagalire era tekukakibwa. Wonna w’owulirira nga oyagala kuvaamu oli waddmebe okuvaamu awatali kukangavulwa konna.

1. **EBIBUUZO KU KUNOONYEREZA KUNO**

Singa obeera n’ekibuuzo kyonna ku kunoonyereza kuno osobola okukwatagana ne Prof. Nelson K. Sewankambo, akulira okunoonyereza kuno eMakerere University College of Health Sciences, P.O. Box 7072, Kampala Uganda: Tel: 0414 530021 oba Muk. Allen Nsangi ne Dr. Daniel Semakula, abamuyambako, ku ssimu 0773333629 oba 0716543000.

Singa obeera n’ekibuuzo kyonna ekikwatagana n’engeri okunoonyerereza mu by’obulamu bwekukolebwa osobola okukwatagana ne Prof. James Tumwine, akulira akakiiko akakwasa empisa muby’okunoonyereza eMakerere, ku ssimu. Tel: 0414 531875.

1. **Okukkiriza kwange**

Ensonga zonna ekikwata kukunoonyereza kuno bazimbuulidde era n’ebibuuzo byange byonna nebiddibwamu. Mpeereddwa obudde obumala okusalawo ku kwetaba mukunoonyereza kuno. Nkitegeera bulungi nnyo nti okussa omukono gwange kukiwandiiko kino tekinzijaako buvunaanyizibwa eri obulamu bwange era tekijaako bantu bakulira kunoonyereza kuno buvunaanyizibwa bwabwe, wabula kiraga nti ensonga zimpeereddwa nsobole okwesalirawo nga omuntu era nzitegedde. Era okusinziira kubimbuuliddwa, nsazeewo neetabe mukunoonyereza kuno nga nzisa omukono gwange ku kiwandiiko kino, wammanga.

| **Nze akkirizza okwetaba mukunoonyereza**  Amannya:_________________________ | Omukono: __________________________ |
| --- | --- |
| Ennaku z’omwezi: __________________ | Essimu: ___________________________ |

| **Abaddewo**  Amannya: __________________________ | Omukono: __________________________ |
| --- | --- |
| Ennaku z’omwezi: ___________________ | Essimu: ___________________________ |
