# Supplementary material for: Can an educational podcast improve the ability of parents of primary school children to assess the reliability of claims made about the benefits and harms of treatments: study protocol for a randomised controlled trial
Source: Trials. 2017 Jan 21;18:31. doi: 10.1186/s13063-016-1745-y (PMC5251251; doi:10.1186/s13063-016-1745-y)
Supplement: Additional file 4: — Setting a standard for the “Claim 12” and “Claim 9”. (DOCX 28 kb) [file 13063_2016_1745_MOESM4_ESM.docx]

**Additional file 4. Setting a standard for the “Claim 12” and “Claim 9”**

The Claim Evaluation Tools are tests that measures an individual’s ability to apply 32 key concepts related to assessing claims about the effects of treatments and making informed healthcare choices. The “Claim 12” includes multiple-choice questions from the Claim Evaluation Tools addressing each of the 12 concepts covered by the IHC primary school resources. The “Claim 9” includes multiple-choice questions from the Claim Evaluation Tools addressing each of the nine concepts covered by the IHC podcast.

It is difficult to interpret average differences in scores on a test or other continuous (or count) outcome measures.^1^ Doing so requires a basis for judging the importance of any differences. In addition, it requires examining the distribution of the scores. For example, a small average difference in test scores might be due to most students doing a little bit better or to a few students doing a lot better than the comparison group.

The difference in the proportion of people who have a passing score is more meaningful and easier to interpret than an average difference in test scores. In this context, passing means:

- Having a basic understanding of the concepts and how to apply them
- Not needing to repeat the lessons, listen to the podcast again, or receive some other additional or alternative instruction
- Being ready to go on to other lessons or another podcast that reinforce learning of the same concepts and introduce new concepts

Determining the proportion of people who pass requires determining a cut-off score, above which someone passes and below which someone does not, or in this context:

- those above the cut-off have a basic understanding of the concepts and are able to apply them, whereas the those below the cut-off do not
- those below the cut-off need to repeat the lessons, listen again to the podcast or receive some other additional or alternative instruction, whereas those above the cut-off do not
- those above the cut-off are ready to go on to other lessons or another podcast, which will reinforce learning of the same concepts and introduce new concepts, whereas those below the cut-off are not

Options for determining a cut-off and the reason for using an absolute (or criterion referenced) standard are summarised in the table below.

**Table A2.1 Options for determining a cut-off**

| **Option** | **Comments** |
| --- | --- |
| Core concepts | This approach bases passing on answering correctly all of the questions that address concepts that are considered core or necessary. Individuals making one mistake would fail. This approach ignores concepts that are not considered “core” and it ignores how difficulty the questions are. |
| Relative standards  (norm referenced) | This approach based on a comparison among the performances of the individuals taking the test. A set proportion of candidates fails. This approach is unfair and does not make sense in the context of evaluating the effectiveness of an educational intervention. |
| Absolute standards  (criterion referenced) | This approach is based on how much an individual knows and is able to apply. Individuals pass or fail depending on whether they meet a specified criterion. |
| Mixed or compromise methods | This approach combines the use of a relative and an absolute approach. It requires a norm (setting a proportion that fail), which does not make sense in the context of evaluating the effectiveness of an educational intervention. |

There are three widely used methods for determining an absolute standard.^2^ All three rely on the concept of individuals who are on the borderline of passing or failing and expert judges. With Nedelsky’s method,^3^ the judges eliminate response options that a borderline individual would be able to eliminate. The chances of getting each question correct is then equal to one divided by the number of remaining response options; e.g. if there are two remaining response options (one of which is the correct option), the chances of a borderline individual answering the question correct is 1/2 or 50%. The cut-off score is then determined by adding up the probabilities for all of the questions.

Angoff’s method is the same as Nedelsky’s,^4^ but the judges judge the difficulty of each question as a whole, instead of making judgements about each response option.

Ebel’s method is similar to Angoff’s,^5^ but judges are asked to make two judgements: one about the relevance of each question and one about the difficulty of each question. They then judge the difficulty of the questions in each cell of a matrix (of levels of relevance by levels of difficulty).

We used a combination of Nedelsky’s and Angoff’s methods. The judges started with Nedelsky’s method, then increased or decreased the assigned probability for each question based on an overall assessment. This gave the judges a logical approach to making an initial judgement about the difficulty of each question. It then allowed them to adjust for uncertainty about the number of response options a borderline individual would eliminate, the difficulty of the stem (scenario) for the question, the difficulty of the concept, and anything else that might make a question more or less difficult.

For each of the above methods there are five steps:

1. Select the judges
2. Define “borderline” knowledge and ability
3. Train the judges in the use of the method
4. Collect their judgements
5. Combine the judgement to choose a passing score

**Selection of the judges**

The judges must be qualified to decide what level of the knowledge or skills measured by the test is necessary to conclude that an individual:

- Has at least a basic understanding of the concepts and ability to apply them
- Does not need to repeat the lessons, listen to the podcast again, or receive some other additional or alternative instruction
- Is ready to go on to other lessons or another podcast, which will reinforce learning of the same concepts and introduce new concepts

Because of the nature of this test and what we are trying to measure, we included two types of judges: health researchers and people who teach evidence-informed decision making, and education researchers with experience evaluating interventions to teach critical thinking skills (Table A2.2). In addition, teachers who participated in pilot testing of the IHC primary school resources reviewed the judgements that are made to ensure they are appropriate for the target audience and the context.

**Table A2.2 Judges**

|  | **Sex** | **Country** | **Background** |
| --- | --- | --- | --- |
| **Judge 1** | M | UK / Kenya | Teacher / health and education researcher |
| **Judge 2** | M | Cameroon / South Africa | Health researcher |
| **Judge 3** | M | UK | Education researcher |
| **Judge 4** | F | USA | Health researcher with PhD in education |
| **Judge 5** | F | UK | Teacher / education researcher |
| **Judge 6** | F | Norway | Health and education researcher |
| **Judge 7** | M | Kenya / Norway | Health researcher |
| **Judge 8** | M | Nigeria / USA | Health researcher |

**Definition of borderline knowledge and ability**

An individual with borderline knowledge and ability is someone who may or may not have a basic understanding of the concepts and ability to apply them, may or may not need additional or alternative instruction, and may or may not be ready to go on to other lessons or another podcast. We created persona who are characteristic of a person with borderline knowledge and ability and of a person who clearly has mastered the concepts. These were used to communicate and help the judges to envisage these people.

**Training of the judges**

We provided the judges with instructions and discuss these with them before they started making their judgements.

The judges took the test before making judgements about the difficulty of the questions. We then gave them the right answers so that they had these when they made their judgements. In this way they got a better sense of how difficult the questions are then if they were given the answers before taking the test themselves.

The judges participated in a practice round with questions that had different degrees of difficulty before they made their individual judgements. This exercise allowed them to discuss what makes a question difficult or easy.

**Collecting the judgements**

The judges independently judged all the questions. Since they were not judging relevance (just difficulty) they could judge all of the questions for the 13 concepts covered in either the Claim 12 or the Claim 9 without having to repeat their judgements for the questions that are used in both Claim 12 and Claim 9.

**Combining the judgements to choose a passing score**

We calculated the mean, median, and trimmed mean for each question and for the cut-off score. We presented all three of these statistics and the range to the judges. We also showed the judges the difficulty of the questions based on the Rasch analysis and discussed any discrepancies in the relative difficulty of the questions between their judgements and the results of the Rasch analysis.^6^ We then asked them to discuss each question and reach a consensus.

We used a modified nominal group approach to reach a consensus.^7^ We first showed everybody all of the judgements for each question. We then invited people from each end of the range to provide the reasons for their judgements, and then invited others to comment. After the final cut-off score was decided, we checked to make sure that all of the judges agreed with the cut-off scores, and adjusted them as necessary, based on a consensus of all eight judges.

**Determining a cut-off score for mastery**

We also asked the judges to make the same set of judgements to set a second cut-off for a score that indicates mastery of the concepts, using the same approach. This cut-off is the minimum score that they would expect for an individual who clearly has a basic understanding of the concepts and ability to apply them, does not need additional or alternative instruction, and is ready to go on to other lessons or another podcast, which will reinforce learning of the same concepts and introduce new concepts.

**Results**

There was substantial disagreement in the judges independent judgements about how difficult each question was. However, there was not substantial disagreement when the probabilities for each question were added up to determine the cut-offs and the judges quickly reached a consensus about both the difficulty of each question and the cut offs. The consensus of all eight judges was that

For the Claim 12, 13 or more questions out of 24 need to be answered correctly to pass and 20 or more questions out of 24 need to be answered correctly to demonstrate mastery.

For the Claim 9, 11 or more questions out of 18 need to be answered correctly to pass and 15 or more questions out of 18 need to be answered correctly to demonstrate mastery.

**References**

1. Guyatt GH, Thorlund K, Oxman AD, Walter SD, Patrick D, Furukawa TA, et al. GRADE guidelines: 13. Preparing summary of findings tables – continuous outcomes. J Clin Epidemiol 2013; 66:173-83.
2. Livingston SA, Zieky MJ. Passing Scores: A Manual for Setting Standards of Performance on Educational and Occupational Tests. Princeton, NJ: Educational Testing Service, 1982.
3. Nedelsky L. Absolute grading standards for objective tests. Educational and Psychological Measurement 1954; 14:(1)3-19.
4. Angoff WH. Scales, norms, and equivalent scores. In: Thorndike RL (ed.), Educational Measurement. Washington, DC: American Council on education, 1971; 514-5.
5. Ebel RL. Essentials of Educational Measurement. Englewood Cliffs, NJ: Prentice-Hall, 1972; 492-4.
6. Austvoll-Dahlgren A, Guttersrud Ø, Oxman AD, et al. Questionnaire to measure people’s ability to assess claims about treatment effects (CLAIM).
7. Jones J, Hunter d. Consensus methods for medical and health services research. BMJ 1995;311:376-80.
